# Comparison of D2 vs D3 lymph node dissection for RIght COloN cancer (RICON): study protocol for an international multicenter open-label randomized controlled trial

**DOI:** 10.1186/s13063-024-08269-5

**Published:** 2024-07-02

**Authors:** Vladimir Balaban, Mikhail Mutyk, Nikolay Bondarenko, Stanislav Zolotukhin, Oleg Sovpel, Igor Sovpel, Dmitriy Zykov, Igor Rublevskiy, Mikhail Klochkov, Alfredo Ponce Prado, Mingze He, Petr Tsarkov

**Affiliations:** 1 G.V. Bondar Republican Cancer Center, Donetsk, Ukraine; 2 M. Gorky Donetsk National Medical University, Donetsk, Ukraine; 3 Sechenov University, Moscow, Russia

**Keywords:** Right colon cancer, D2 lymph node dissection, D3 lymph node dissection

## Abstract

**Background:**

Colon cancer is a global health concern, ranking fifth in both new diagnoses and deaths among tumors worldwide. Surgical intervention remains the primary treatment for localized cases, with a historical evolution marked by a focus on short-term outcomes. While Japan pioneered radical tumor removal with a systematic categorization of lymph nodes (D1, D2, D3), the dissemination of Japanese practices to the West was delayed until 90th of last century. Discrepancies between Japanese D3 dissection and the CME with CVL principle persist, with variations in longitudinal margins and recommended procedures. Non-randomized trials indicate the superiority of D3 over D2, but a consensus is lacking.

**Methods:**

This prospective, international, multicenter, randomized controlled trial employs a two-arm, parallel-group, open-label design to rigorously compare the 5-year overall survival outcomes between D2 and D3 lymph node dissection in stage II-III right colon cancer. Building on prior studies, the trial aims to address existing knowledge gaps and provide a comprehensive evaluation of the outcomes associated with D3 dissection. The study population comprises patients with right colon cancer, ensuring a focused investigation into the specific context of this disease. The trial design emphasizes its global scope and collaboration across multiple centers, enhancing the generalizability of the findings.

**Discussion:**

This study’s primary objective is to elucidate the potential superiority in 5-year overall survival benefits of D3 lymph node dissection compared to the conventional D2 approach in patients with stage II-III right colon cancer. By examining this specific subset of patients, the research aims to contribute valuable insights into optimizing surgical strategies for improved long-term outcomes. The trial’s international and multicenter nature enhances its applicability across diverse populations. The outcomes of this study may inform future guidelines and contribute to the ongoing discourse surrounding the standardization of colon cancer surgery, particularly in the context of right colon cancer.

**Trial registration:**

ClinicalTrials.gov NCT03200834. Registered on June 27, 2017.

**Supplementary Information:**

The online version contains supplementary material available at 10.1186/s13063-024-08269-5.

## Background and rationale

Colon cancer ranks 5th globally in both the number of newly diagnosed cases and the number of deaths among tumors in other locations [1]. The primary treatment method for patients with localized forms of colon cancer is surgery [2]. Lymphatic outflow from the right colon was described by Jamieson in 1909. In that study, regional lymph nodes were classified as paracolic, intermediate, and main nodes [3]. Nevertheless, the removal of all three levels of regional lymph nodes was not described in the literature as routine practice. Later, in Japan, questions regarding radical tumor removal began to emerge [4]. These questions primarily focused on the removal of the primary tumor and all regional lymph nodes, which were categorized into paracolic intermediate and apical nodes. Lymph node removal was classified as D1 (paracolic), D2 (paracolic and intermediate), and D3 (paracolic, intermediate, and apical). For stage II-III colon cancer, D3 lymph node dissection becoming mandatory [5].

However, it is worth noting that publications from Japan only became available to Western countries after 1990, leading to discussions in the West commencing in 2003 [6]. Subsequently, an article was published in 2009 regarding the standardization of colon cancer surgery [7], which introduced the abbreviation CME with CVL (complete mesocolic excision with central vascular ligation). Despite the prevalence of Japanese D3 lymph node dissection and the CME with CVL principle, there are certain differences [8]. Longitudinal margins with D3 lymph node dissection involve segmental resections of the colon depending on the feeding artery of the tumor, while an alternative method entails maintaining a 10-cm distance in the proximal and distal directions from the tumor. In the European approach, hemicolectomy or subtotal colectomy is recommended [6, 7]. The fundamental concept in D3 lymph node dissection is the removal of all regional lymph nodes, while in the CME with CVL concept, it involves sharp dissection with preservation of the mesocolic fascia and high ligation of the feeding vessels. The primary objective of both techniques is to enhance overall and disease-free survival. Numerous non-randomized controlled trials have demonstrated the superiority of D3 lymph node dissection over D2 in terms of overall and disease-free survival [9–12]. Other authors have shown comparable long-term results [13, 14]. Currently, the RELARC trial has investigated long-term outcomes (primary endpoint being 3-year disease-free survival) for right colon cancer, although the gold standard for treatment effectiveness remains 5-year overall survival [15]. In ins turn, COLD trial looked into 5-year overall survival between D2 and D3 lymph node dissection, but it included both right and left colon cancer as well as stage IV of the disease [16].

## Objectives

The aim of this study is to compare the 5-year overall survival between D2 and D3 lymph node dissection for right colon cancer in patients with stage II-III of the disease.

## Trial design

**Fig. 1:**
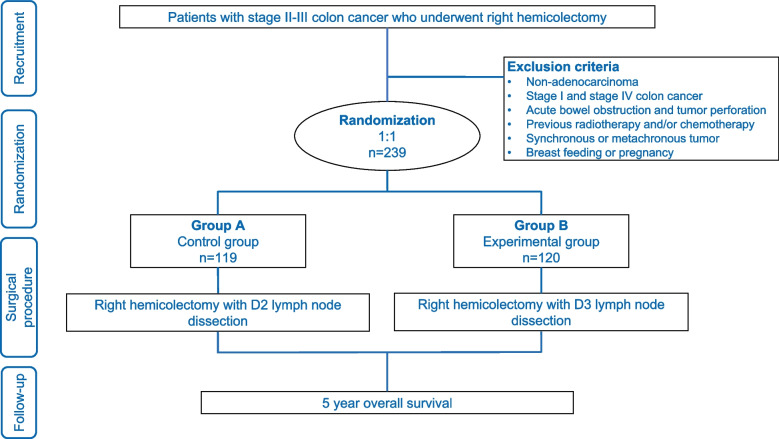
Flowchart of the study design

The study is an international, multicenter, two-arm, parallel-group, open-label, randomized controlled trial designed to ascertain whether D3 lymph node dissection confers a 5-year overall survival superior than D2 lymph node dissection (Fig. 1). We must address why there was a delay in submitting the study protocol. The primary reason stems from our initial uncertainties surrounding our ability to meet the large sample size requirement for recruitment, which posed a significant challenge for a single clinical center. In light of this, we expanded our network to include additional centers, thereby transitioning the study from a single-center to a multicenter trial.

## Methods

### Study setting

**Fig. 2:**
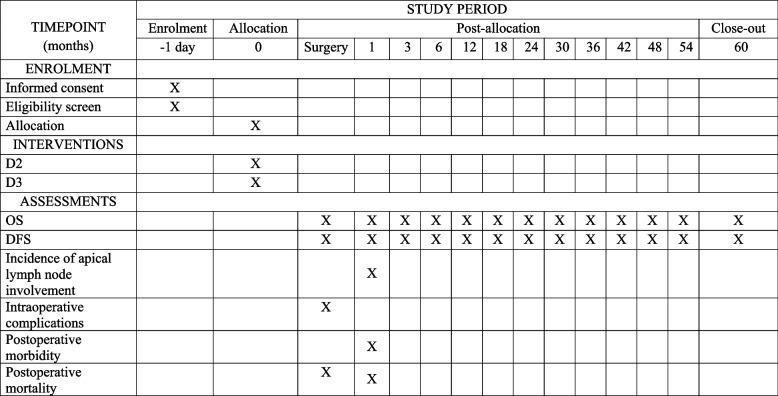
SPIRIT-figure

Patients will be recruited from the Departments of Colorectal Cancer Surgery #1 and #2, as well as the Department of Laparoscopic and Minimally Invasive Surgery at the G.V. Bondar Republican Cancer Center in Donetsk, Ukraine. Additional recruitment locations include the Clinic of Coloproctology and Minimally Invasive Surgery at Sechenov University, Moscow City Oncology Hospital No. 62 in Moscow, Russia, and St. Luke’s Clinical Hospital in St. Petersburg, Russia (Fig. 2). All patients who meet the eligible criteria are proposed to be recruited in all centers. This study protocol is documented in accordance with the SPIRIT reporting guidelines [17]. The results of the trial will be reported in the subsequent publications.

### Reference to the clinic where the list of study sites can be obtained

- https://www.rusprofile.ru/id/1229300085387 —G.V. Bondar Republican Cancer Center
- https://sechenovclinic.ru/hospitals/detail.php?id=614 —Sechenov University
- https://onco62.ru —Moscow City Oncology Hospital No. 62
- https://lucaclinic.ru —St. Luke’s Clinical Hospital

### Randomization and blinding

A simple 1:1 randomization was used in this study. A random number list was build using R studio software by an independent statistician and downloaded to electronic case report form (eCRF) with an allocation ratio 1:1 (D2 and D3 groups). After carefully selecting patients based on the inclusion and exclusion criteria, informed consent will be obtained by the corresponding investigators and the patients will be assigned to the corresponding intervention group. The trial is open-label, the surgeons are unblinded, and patients are clearly informed about the procedures they will undergo, as required by the healthcare systems of all centers in both countries. In the D2 lymph node dissection group, in case of intraoperative macroscopically suspicious apical lymph nodes are observed, the surgeon should remove them for ethical reasons. The patient will not be transferred to the D3 lymph node dissection group and will continue with follow-up as per the D2 group.

### Study population and eligible criteria

#### Inclusion criteria

1. Provision of signed informed consent by patients
2. Confirmation of adenocarcinoma of the right colon (cecum, ascending, hepatic flexure, and proximal transverse colon) verified by colonoscopy
3. Tumor staging as cT_3-4a,b_N_0_M_0_ (stage II) or cT_1-4a,b_N_1-2_M_0_ (stage III) on CT
4. Demonstrated tolerability of chemotherapy drugs
5. Age ranging from 18 to 75 years
6. American Society of Anesthesiologists (ASA) physical status classification of 1–3

#### Exclusion criteria

1. Presence of distant metastases (cM1)
2. Tumor staging as cT_is_–cT_2_N_0_ (stage I) and cT4_b_ (involving the liver, kidney, head of the pancreas and duodenum, vena cava, aorta, or superior mesenteric vessels)
3. Emergent cases (limited to tumor perforation and acute bowel obstruction)
4. History of previous chemotherapy or radiation therapy
5. Presence of synchronous or metachronous cancer
6. Pregnancy or breastfeeding
7. Refusal to participate in trial

#### Withdrawal criteria

Exploratory laparotomy/laparoscopy or other reasons for refusing resection.

#### Criteria for study centers and intervention performers

1. The hospital must specialize in colorectal cancer surgery.
2. Surgeons involved in the study should have experience in conducting a minimum of 50 elective colon cancer resections.
3. They must demonstrate a commitment to fully complying with the protocol requirements of this study.

### Interventions

#### Informed consent

Doctors will present the trial to patients and provide them with information cards. They will then discuss the details of the trial as outlined on the cards with the patients. Following these discussions, doctors will seek written consent from patients who agree to participate in the trial.

#### Laboratory research

1. Complete blood count (CBC) and urine tests
2. Biochemical blood tests (urea, creatinine, bilirubin, blood sugar, ALT, AST, α-amylase, total protein)
3. Coagulogram
4. Tumor markers: CEA, CA19-9

#### Instrumental studies

1. Colonoscopy with biopsy for diagnosis verification
2. Computed tomography of the abdominal and thoracic cavity with intravenous bolus contrast
3. Esophagogastroduodenoscopy
4. Electrocardiogram, echocardiography if necessary

#### Surgery

The surgical procedures encompass two types: right hemicolectomy and extended right hemicolectomy. The distinction lies in the level of middle colic artery ligation and the removal of different portions of the transverse colon. In right hemicolectomy, only the right branch of the middle colic artery is transected, along with division of the proximal transverse colon. Extended right hemicolectomy involves ligation of the middle colic artery at its origin and transection of the mid transverse colon.

#### Techniques for the right *colon* mobilization

The surgical technique for the right colon mobilization includes four steps: cranial, caudal, medial, and lateral. The cranial approach initiates from entry into the lesser sac and continues distally to the level of ileocolic pedicle. The caudal mobilization starts from the embryonic plane under the ileum and progresses proximally to the level of the Henle’s trunk. The medial approach involves movement into the embryonic plane beneath the ileocolic artery and continues in the proximal, distal, and lateral directions to the levels of transverse colon and ileum transection. Lateral approach is carried out by dissecting Toldt’s line between the mesocolic fascia and Gerota’s fascia, continuing until the level of superior mesenteric vessels. Full-length mobilization includes the preservation of mesocolic fascia, except the cases of locally advanced cancer, where excision beyond the embryological planes is required.

#### The boundaries of D2 and D3 lymph node dissection

The proximal boundary of D2 lymph node dissection is defined by the location of mesocolon fixation to the Gerota’s fascia and the descending duodenum. In D3 lymph node dissection, the additional boundary extends to the head of the pancreas, up to the gastroepiploic vein and the lower edge of the pancreatic neck.

The distal boundary for D2 lymph node dissection starts from the ileum, 10 cm from the cecum, continuing 1 cm below the projection of ileocolic vessels until 1 cm laterally from the superior mesenteric vein (SMV). For D3 lymph node dissection, the distal resection margin additionally includes the opening of the anterior surface of the SMV, 1 cm below the origin of ileocolic vein. Paracolic lymph nodes should be removed at a minimum distance of 10 cm from the tumor in both the proximal and distal direction.

The lateral border for D2 and D3 lymph node dissection is defined by the white line of Toldt.

The surgical trunk (medial boundary) for D2 lymph node dissection is set at 1 cm from the lateral edge of the SMV at the level of the ileocolic artery and vein, extending proximally to the anterior surface of the duodenum. The head of the pancreas and origin of the feeding arteries are not visualized, along with the superior mesenteric vein and Henle’s trunk.

**Fig. 3:**
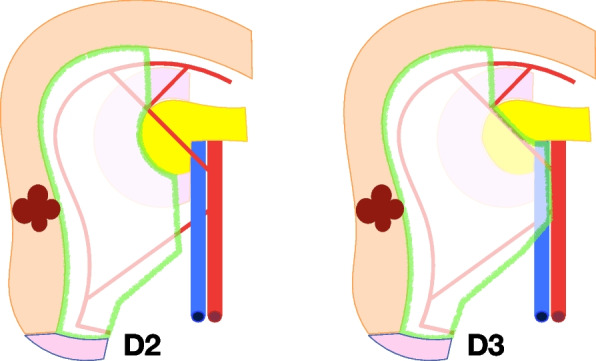
Schematic representation for right hemicolectomy with D2 and D3 lymph node dissection. The green area indicates the mesentery that underwent removal

**Fig. 4:**
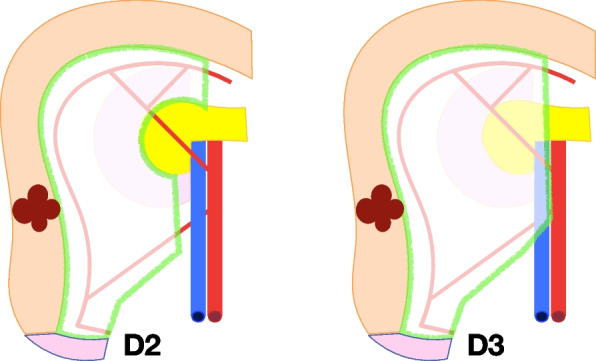
Schematic depiction for extended right hemicolectomy with D2 and D3 lymph node dissection. The green area signifies the mesentery subjected to removal

**Fig. 5:**
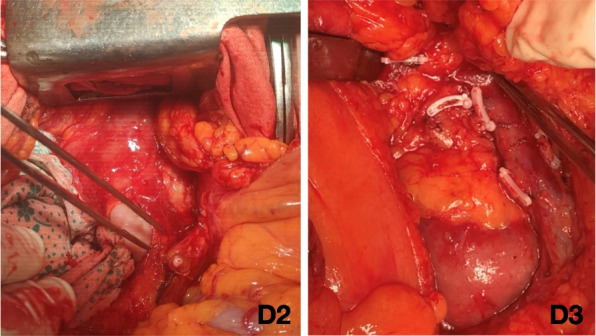
Intraoperative view for open D2 and D3 lymph node dissection

**Fig. 6:**
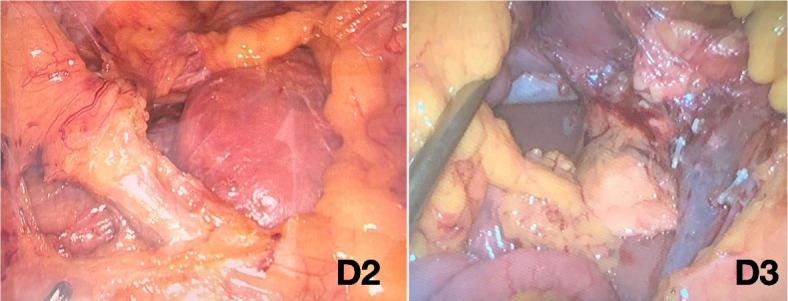
Intraoperative view for laparoscopic D2 and D3 lymph node dissection

The surgical trunk (medial boundary) for D3 lymph node dissection is the medial side of the SMV from the lower border of the pancreatic neck to 1 cm below the origin of the ileocolic vein (Figs. 3, 4, 5, and 6). The anterior surface of the SMV, origin of the feeding vessels, and ligation of the intestinal tributary of Henle’s trunk serve as anatomical landmarks of complete D3 lymph node dissection. When the ileocolic artery and/or right colic artery pass behind the SMV, the ligation of these arteries is carried out at the lateral side of the SMV.

For hepatic flexure or proximal transverse colon cancer and suspicious infrapyloric and/or greater curvature lymph nodes, D3 lymph node dissection may include its removal.

#### Concomitant care

Routine concomitant care is consistent between the two groups. No specific or prohibited interventions or requirements have been identified for this trial.

#### Photographic recording and quality assessment of the removed specimen

Following the application of the clips and before crossing the vessels, a photo of the surgical field is captured to assess the compliance of the performed lymph dissection with the declared procedure.

**Fig. 7:**
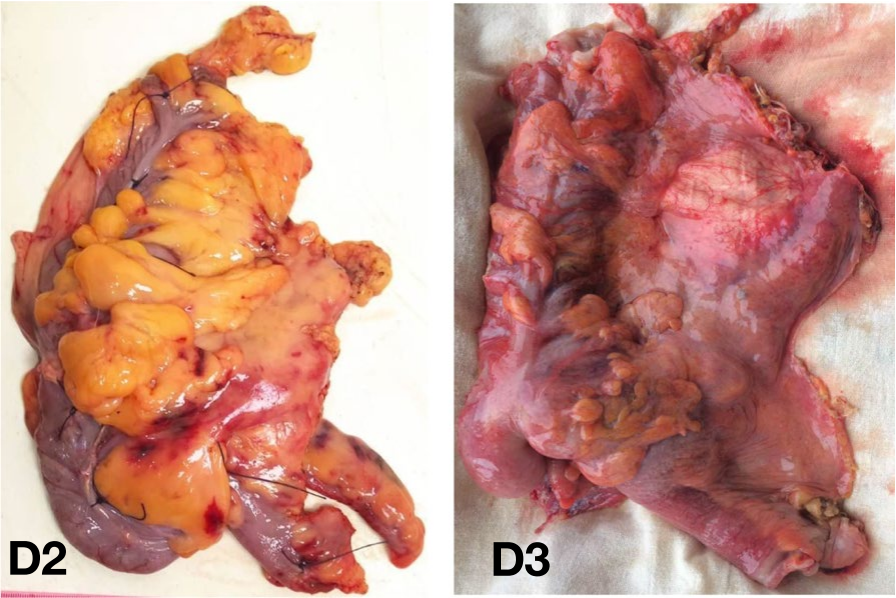
Photograph of the specimen after D2 and D3 lymph node dissection (intraperitoneal view)

**Fig. 8:**
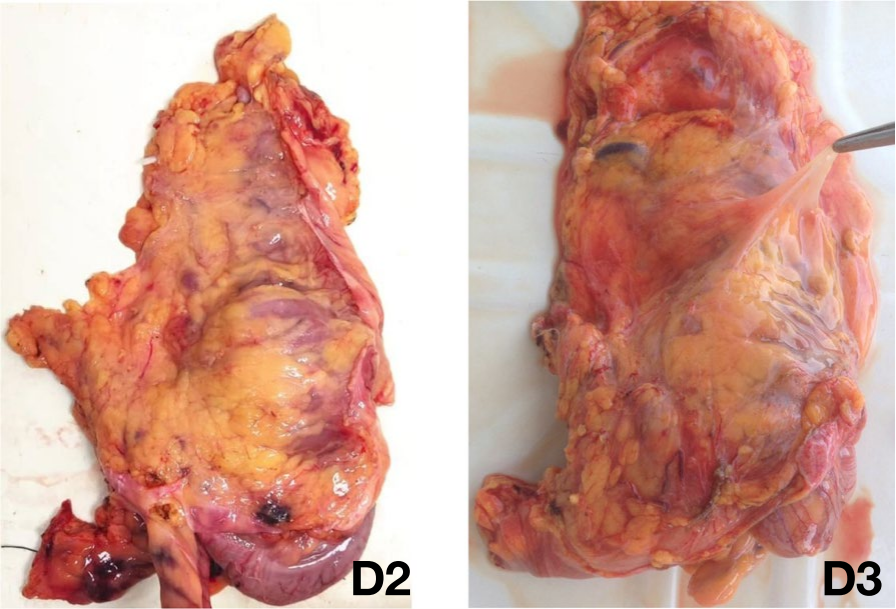
Photograph of the specimen after D2 and D3 lymph node dissection (extraperitoneal view)

Photographic recordings of the specimen are taken on both sides (intraperitoneal and retroperitoneal) to assess the preservation of the mesocolic fascia (Figs. 7 and 8).

The quality of the specimen is determined according to West classification [18], and this evaluation is conducted independently by a collaboration of the surgeons and pathologists.

#### Specimen preparation for pathologists

**Fig. 9:**
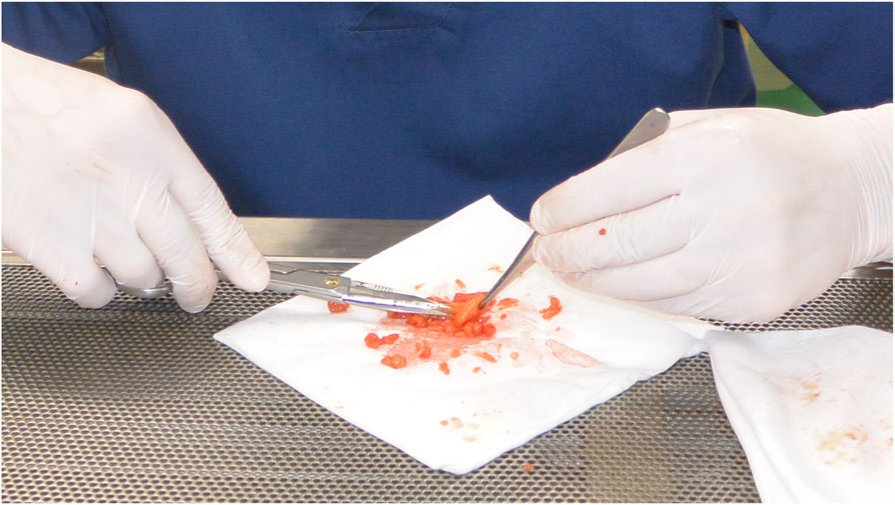
Extraction of lymph nodes from the specimen

Lymph node extraction from the fresh specimen is performed by surgeons after photographic recording of the specimen on both sides (Fig. 9). All mesentery is meticulously separated into groups of lymph nodes. An exception is made for the area of the paracolic mesentery, which is limited by the size of the tumor. This exception is intended to determine tumor invasion into the mesentery, as well as extramural invasion, tumor deposits, tumor budding, and circular resection margin (CRM).

**Fig. 10:**
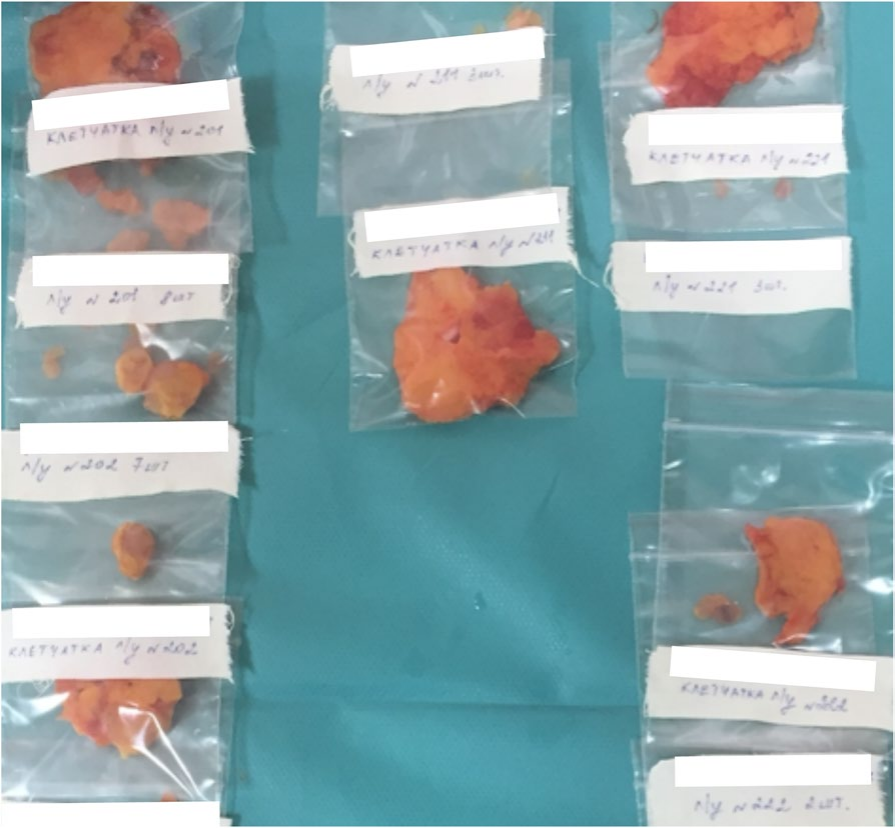
Final view for the pathologist after lymph node extraction

The mesentery of the colon is dissected, and all visible lymph nodes are well isolated. Separate containers with selected lymph nodes, distributed by each group, along with the mesentery from which they were extracted (for additional detection of lymph nodes and other findings), will be sent to the pathologist (Fig. 10).

#### Specimen controlling process

The material was fixed for 12 h in 10% neutral formalin, after which the material was cut out by a pathologist according to the standard protocol. Selected fragments undergo standard dehydration process in the pathological departments from all centers. Several sections (2–4) with a thickness of 5–6 μm were formed from the paraffin blocks. Hematoxylin and eosin were used for staining. The prepared slides were examined by a pathologist under a microscope.

All macroscopic specimens collected will be stored in a 10% solution of neutral formaldehyde until the trial finished.

After finishing of the trial, sealed disposable containers with all collected specimens are used in agreement with the State Sanitary Inspection Regulation for medical institutions or in a centralized way (thermal neutralization—cremation).

#### Adjuvant chemotherapy

Adjuvant chemotherapy will not be administered for stage I of the disease or stage II without risk factors such as poor tumor differentiation, extramural invasion, intestinal obstruction, tumor perforation during surgery, less than 12 examined lymph nodes, or involvement of resection margins. In all other cases, a fluoropyrimidine and/or oxaliplatin regimen will be employed.

#### Follow-up

The entire follow-up process will be meticulously monitored by the investigators involved. Regular contact with patients will be established to implement the necessary follow-up strategies.

1. Abdominal ultrasound every 3 months for the first 2 years, then every 6 months until the 5th year
2. CEA monitoring every 3 months for the initial 2 years, then every 6 months until the 5th year
3. Chest and abdomen CT scans every 6 months during the first 2 years, then annually until the 5th year
4. Colonoscopy every year for 5 years postoperatively

### Outcome parameters

#### Primary endpoint

A 15% increase in 5-year overall survival (OS) utilizing D3 lymph node dissection.

#### Secondary endpoint

1. 5-year disease-free survival (DFS)
2. Incidence of apical lymph node involvement
3. Intraoperative complications
4. Postoperative morbidity
5. Mortality

### Definition of endpoints

#### OS and DFS

OS is the proportion of individuals in a study (in each group) who are alive 5 years after receiving the intervention. OS is defined by any-cause mortality.

DFS is the proportion of individuals in a study (in each group) who are alive 5 years after receiving the intervention, without experiencing local or distant recurrence or death from any cause. For DFS, an event is defined as local or distant recurrence or death. Participants who are lost to follow-up during the 5-year period post-procedure will be classified as censored. The survival analysis will be conducted using Kaplan–Meier curves, with comparisons made using the log-rank test.

#### Incidence of apical lymph node involvement

The incidence of apical lymph node involvement is the histologically confirmed percentage of patients who underwent D3 lymph node dissection with positive lymph nodes.

#### Intraoperative complications

All intraoperative complications will be diagnosed during the operation.

1. Vascular injury: An unintentional full-thickness defect of the vessel (arteries and veins) wall caused by surgical manipulations. Arterial vessels include the ileocolic, right colic, middle colic, accessory middle colic, gastroepiploic, superior mesenteric, and aorta. Venous vessels include the ileocolic, right colic, gastrocolic trunk and its tributaries, middle colic, superior mesenteric, and cava.
2. Tumor rupture/perforation: An unintentional partial or full-thickness defect of the colon wall where the tumor is located, caused by surgical manipulations.
3. Abscess perforation: An unintentional defect in the abscess capsule accompanied by the release of pus into the abdominal cavity.

The method of aggregation for all intraoperative complications will involve calculating the proportion of patients who experience complications during the operation relative to the total number of patients. Group comparisons will be conducted using the chi-square or Fisher’s exact test.

#### Postoperative morbidity

All postoperative morbidity will be diagnosed within 30 days after the procedure, except for postoperative diarrhea.

1. Postoperative lymphatic leakage: Includes lymphatic fistula, lymphorrhea, lymphocele, lymphatic ascites, and special forms (chylous leakage), classified according to the Lv classification [19].
2. Anastomotic leakage: A defect of the intestinal wall at the anastomotic site leading to a communication between intra- and extraluminal compartments. The severity is graded based on clinical management impact. Grade A anastomotic leakage results in no change in patients’ management, whereas grade B leakage requires active therapeutic intervention but is manageable without re-laparotomy. Grade C anastomotic leakage requires re-laparotomy [20].
3. Postoperative diarrhea is loose stool, watery stool, or mucous stool three times or more a day within 6 months after the operation.

The aggregation method for all postoperative morbidities will involve calculating the proportion of patients who experience complications within the first 30 days post-operation relative to the total number of patients. Group comparisons will be conducted using the chi-square or Fisher’s exact test.

#### Postoperative mortality

Postoperative mortality is defined as death from any cause within the first 30 days following the operation. It will be measured by the proportion of deceased patients relative to the total number of patients. Group comparisons will be conducted using the chi-square or Fisher’s exact test.

### Sample size calculation

According to the SEER database (2004–2012) and AJCC 5th, 6th, and 7th editions, the 5-year overall survival for stage II right colon cancer was 68–84%, and for stage III, it was approximately 57 to 60% [21–23]. In the JCOG0404 trial, the 5-year overall survival for stage II-III was 91% [24]. Literature data suggests that the baseline survival for D2 lymph node dissection group is estimated to be 75% for stage II-III of the disease, and for D3 group, it is 90%, respectively. An anticipated difference of 15% in 5-year overall survival between D2 and D3 LND groups is expected. The log-rank test (Lacatos, proportion of surviving) was utilized in the PASS 11 program for sample size calculation. The power of the study is set at 80%, with a type I error 5%. An expected loss of follow-up is estimated at 15% for patients in each group. The enrollment period for patients is 3 years, and the total duration of the study will be 8 years. Based on aforementioned conditions, 239 patients should be included in the study (Table 1). Ultimately, 120 patients will be included in D3 group, and 119 patients in D2 group. The anticipated number of deaths in the D2 group is 24, and in the D3 group, it is 10.

**Table 1:**
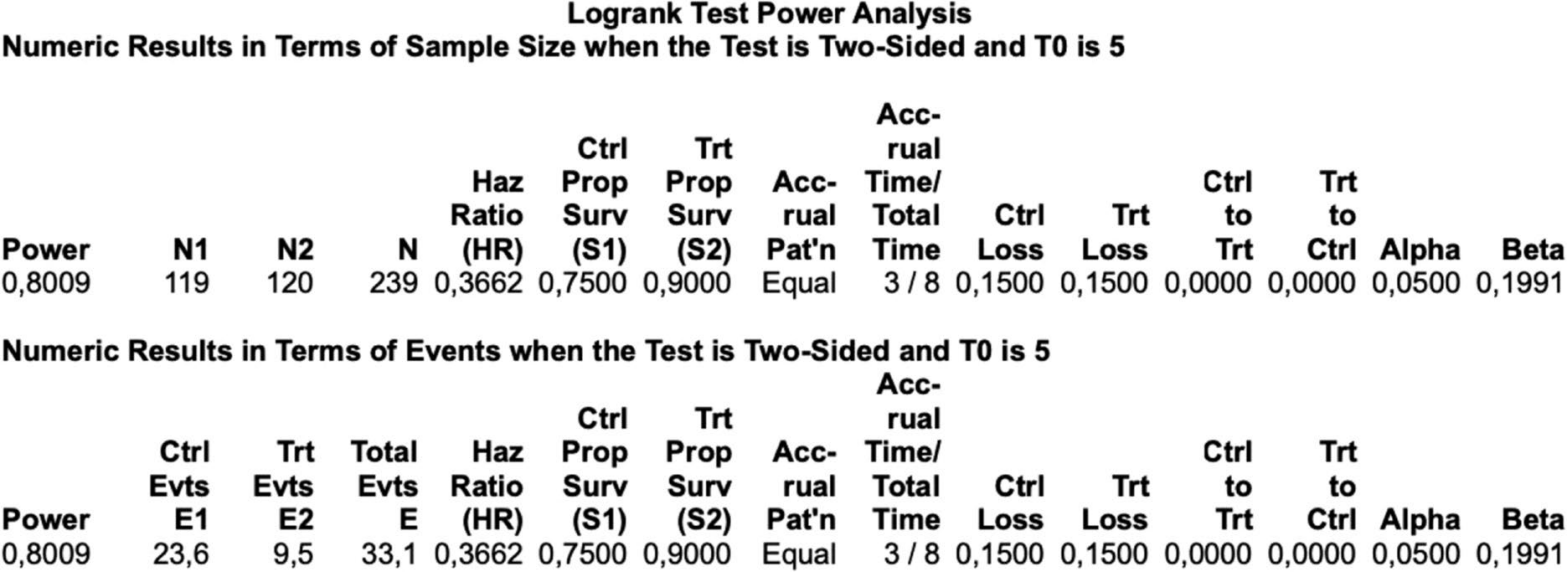
Sample size calculation

### Data analysis

Upon recruiting the last patient, a 5-year follow-up will commence. Intention-to-treat and per-protocol analysis will be conducted. Missing data will be imputed using Multivariate Imputation by Chained Equations (MICE package in R), except for survival data [25]. All collected data will be meticulously stored in the eCRF, and subsequent statistical analysis will be conducted. The data assessment process will be conducted in an anonymized manner, controlled by members of the university’s research group, who will solely handle the collected data. Data analysis will be solely conducted by the statisticians, also in an anonymized manner. The numeric variables will be presented as mean and standard deviation or median and range. For categorical variables, numbers and percentages will be expressed. *T*-test or Mann–Whitney *U* test will be employed for comparison between numeric variables, and chi-square or Fisher’s exact test will be used to compare categorical variables. A subgroup analysis by stage of the disease will be conducted. The survival analysis will be estimated using Kaplan–Meier curves, and comparisons will be made using the log-rank test. Cox regression will be applied to analyze the risk factors of survival. The event for overall survival will be defined as death from any cause, and for disease-free survival, it will be local or distant recurrence or death. Patients lost to the 5-year follow-up will be censored. All statistical tests are two-sided, and statistical significance is defined as *p* < 0.05. The statistical analysis will be performed in R Studio under the Affero General Public License version 3.

### Data monitoring

An independent data monitoring committee (DMC) has been established, comprising experienced colorectal surgeons, biostatisticians, research contractors, and ethicists. A specifically assigned independent specialist will run the assessment of intraoperative field and re-evaluation process and handle the post-operative specimens according to the study design and protocol requirements. After surgery, all post-operative complications and events will be monitored by a specific physician and recorded in the eCRF. For data collection, additional specialists will be assigned who will not be involved in the perioperative management. Confidentiality agreements have been signed by all investigators involved to protect the personal information of the participants. All study-related information will be stored securely at the study site. The DMC members will convene before the commencement of the study and at regular intervals throughout its duration. The committee will assess various aspects of the study, including adverse events, participant withdrawals, and endpoints. The finial dataset will be assessed by all involved investigators and all members of DMC. The auditing will be conducted once per year and the process will be independent from investigators and the sponsor. In the event that any major changes arise during the conduct of the trial, all involved parties will convene a meeting to address potential concerns. The involved data will be stored securely for a period of 5 years after enrollment of the last participant.

### Reporting of postoperative complication

All potential harms will be collected systematically. MedDRA will be used to classify any potential harms, and all harms will be reported according to the Clavien-Dindo classification [26].

### Ethics and research registration

Comparison of D2 vs D3 lymph node dissection for right colon cancer (RICON NCT03200834) was approved by the Local Ethics Committee of Donetsk National Medical University under reference No. 24/1 dated 01/20/2017.

### Interim analysis and monitoring

An interim analysis will be conducted once, considering multiplicity using the Lan–DeMets method with O’Brien and Fleming type boundaries. The DMC will independently review the interim analysis report and prematurely terminate the study in the interest of patient safety and well-being if necessary.

## Discussion

This study is the first randomized controlled trial to evaluate the effectiveness of D3 lymph node dissection exclusively for right colon cancer based on 5-year overall survival. In the study, we intend to apply the experience in treating of right colon cancer from Japan to the East Slavic population with minor adjustments in surgical intervention. Our trial proposes the performance of only hemicolectomy, with the exception of segmental resections. The removal of infrapyloric and greater curvature lymph nodes will not be mandatory in this study. All other conditions, such as the isolation of lymph nodes on a fresh specimen, will be fulfilled.

According to the evidence-based recommendations for blinding in surgical trials [27], this study is a surgical-based RCT rather than a pharmacological one. Therefore, performance biases are impossible to exclude in this trial because patients and surgeons cannot be blinded. Patients should be clearly informed about the procedures they will undergo, as required by the healthcare systems of all centers in both countries. The primary endpoints will not be compromised by the procedures provided. All possible procedure options will be stated in the informed consent agreement without indicating the superiority of any procedure to all patients.

Additionally, the primary endpoint of this study will not be influenced by the surgeons, because a specifically assigned independent specialist will run the assessment of intraoperative field and re-evaluation process and handle the post-operative specimens according to the study design and protocol requirements. After surgery, all post-operative complications and events will be monitored by a specific physician and recorded in the eCRF. For data collection, additional specialists will be assigned who will not be involved in the perioperative management. The detection biases will not be compromised because each group of participants will be independent of each other.

The data assessment process will be conducted in an anonymized manner, controlled by members of the university’s research group, who will solely handle the collected data. Data analysis will be solely conducted by the statisticians, also in an anonymized manner.

The choice of surgical procedure, such as right hemicolectomy or segmental resection, basically depends on the proximal and distal resection margins. This decision is intricately linked to assessing the risk of paracolic lymph node involvement. Based on studies conducted on the Japanese population, 10 cm in the proximal and distal directions is considered optimal [28, 29]. In our study, we are performing hemicolectomy or extended hemicolectomy to investigate the risk of lymph node involvement in the East Slavic population, within a distance of 10 cm or more from the tumor.

There is no universally accepted consensus regarding the routine removal of infrapyloric and greater curvature lymph nodes for cancer of the hepatic flexure and transverse colon. On the one hand, these lymph nodes are derived from the dorsal mesogastrium and mesoduodenum [30, 31] and are classified as M1 according to the TNM classification for colon cancer. On the other hand, the incidence of involvement of these lymph nodes can vary from 0.7 to 11% [32, 33]. In our study, lymph node dissection of these nodes will only be performed if suspicion arises.

According to the Japanese classification of lymph nodes, the apical lymph node is located along the arterial vessels. From the 1970s to the 1990s in Japan, lymph node dissection for right colon cancer was performed along the superior mesenteric artery (SMA). However, in 1991, a cadaveric investigation conducted by Sato demonstrated that the lymphatic flow from the right colon follows the SMV instead SMA [34]. One of the first papers published in English by Toyota et al. in 1995 described the medial border of D3 lymph node dissection along the anterior surface of the SMV [29]. Currently, the medial border of D3 lymph node dissection for the right colon cancer still follows the SMV [35]. Nevertheless, the Japanese classification still demonstrates apical lymph nodes along the origin of the feeding artery, which is the SMA. Continued investigations into lymphatic flow have indicated that lymphatic vessels and lymph nodes are located not only around the SMV but also the SMA [36]. Furthermore, recent data from Japan demonstrate involvement of lymph nodes around the SMA, especially for T4 tumors [37]. In our study, the anterior surface of the SMV was selected as the surgical trunk due to new information about lymphatic outflow and the incidence of SMA lymph node involvement that emerged after the trial had begun.

The posterior location of the ileocolic artery (behind the SMV) is more common in the Russian population compared to the Chinese [38]. Lymph node dissection of apical lymph nodes remains a controversial issue, especially in patients with an ileocolic artery course behind the SMV. Thus, Spasojevic suggests removing tissue under the SMV when the ileocolic artery is located posteriorly. However, Numata demonstrates the intersection of the ileocolic artery with its posterior location relative to the SMV at its lateral edge [39]. In the RICON study, ligation of the ileocolic artery was performed at the lateral edge of the SMV, as performed by most surgeons in Japan.

One of the main challenges in lymph node dissection studies is the heterogeneity in terminology. When discussing the medial border of lymph node dissection for the right colon cancer, the concepts of D3 and CME include the removal of adipose tissue above the anterior surface of the SMV [7, 29]. The medial border during D2 lymph node dissection and non-CME shows no similarities in anatomical landmarks. The term non-CME should be avoided because any resection, such as D0, D1, D2, or any other, that differs from CME can be titled as non-CME. This can be misleading when interpreting treatment results. There is no precise definition of the medial border of D2 lymph node dissection in the literature. In some studies, the medial border of D2 lymph node dissection is considered to be the lateral edge of the SMV [16, 40, 41], while others describe D2 lymph node dissection as a conventional right colectomy [42]. In our study, the medial border of D2 lymph node dissection was closer to the terminology used by Balciscueta. This decision was also influenced by the absence of randomized trials comparing D2 or conventional lymph node dissection with D3 lymph node dissection. In our view, the evidence for the effectiveness of D3 lymph node dissection should stem from significant differences in the extent of lymph node dissection. This is one of the explanations for the 15% difference we chose for sample size calculation. In our trial, the surgical approaches differed highly than that in the RELARC trial. Thus, the effect size between these trials is different. The RELARC trial focused on the small effect size because the difference in surgical approach was superior mesenteric vein dissection [15]. Consequently, according to the effect sizes in biomedical trials reported by Christopher et al. [43], the RELARC trial aims to identify a small effect size, while the RICON trial aims to identify a medium effect size. It is encouraging that studies examining lymph node dissection techniques with less obvious differences in overall and disease-free survival are running parallel to our study [16, 40]. This collective body of research will contribute valuable insights into the effectiveness of different lymph node dissection options in the future.

## Trial status

### Protocol version

Date: 12 Oct. 2023

Version: 1.3

Date recruitment began: 29 June 2017

Date recruitment finished: 11 Sep. 2023

### Supplementary Information

Additional file 1. SPIRIT checklist.

## Data Availability

The data that support the findings of this study are not openly available due to reasons of sensitivity and are available from the corresponding author upon reasonable request.

## Abbreviations

CME with CVL: Complete mesocolic excision with central vascular ligation
eCRF: Electronic case report form
CBC: Complete blood count
SMV: Superior mesenteric vein
OS: Overall survival
DFS: Disease-free survival
DMC: Data monitoring committee
SMA: Superior mesenteric artery
CRM: Circular resection margin
